# Erectile Function Before and After Elective Inguinal Hernia Repair: A Paired IIEF-5 Study Comparing Laparoscopic and Open Techniques

**DOI:** 10.3390/jcm15124433

**Published:** 2026-06-08

**Authors:** Volkan Sayur, Mehmet Avcı, Erkan Güler, Berk Can Karabağ, Taylan Özgür Sezer

**Affiliations:** 1 Department of Surgery, School of Medicine, Ege University, İzmir 35080, Turkey; volkan.sayur@ege.edu.tr (V.S.); mavciii@hotmail.com (M.A.); drberkcankarabag@gmail.com (B.C.K.);; 2 Department of Surgery, School of Medicine, Mersin University, Mersin 33110, Turkey

**Keywords:** erectile function, IIEF-5, inguinal hernia, laparoscopy, scrotal hernia

## Abstract

**Objective:** This study aimed to evaluate changes in erectile function, assessed using the International Index of Erectile Function-5 (IIEF-5), in male patients undergoing elective inguinal hernia repair and to compare outcomes according to surgical technique and hernia characteristics. **Materials and Methods:** A total of 126 male patients who underwent elective unilateral inguinal hernia repair using either laparoscopic transabdominal preperitoneal/totally extraperitoneal repair or open Lichtenstein repair were included. Patients with severe comorbid conditions that could affect erectile function or surgical outcomes were excluded. Preoperative erectile function was assessed using the IIEF-5 questionnaire during routine preoperative evaluation within one month before surgery, and postoperative assessment was performed using the same questionnaire at the sixth postoperative month. Routine tacker fixation was used in all laparoscopic procedures. Subgroup analyses were performed according to the presence of scrotal hernia, and independent predictors of postoperative change in IIEF-5 score were evaluated using multivariate linear regression analysis. **Results:** Laparoscopic repair was performed in 59 patients (46.8%), whereas 67 patients (53.2%) underwent open Lichtenstein repair. In the overall cohort, the mean IIEF-5 score increased from 22.85 ± 3.12 preoperatively to 23.35 ± 1.98 at the sixth postoperative month (*p* < 0.05). No significant difference in IIEF-5 score change was observed between laparoscopic and open repair groups (*p* > 0.05). Although the overall postoperative increase in IIEF-5 score was statistically significant, its magnitude was small. Patients with scrotal hernia showed a greater postoperative IIEF-5 score increase compared with those without scrotal hernia (*p* < 0.001), but this subgroup was small. In multivariate analysis, scrotal hernia status (β = +2.41; *p* = 0.041) and lower baseline IIEF-5 score (β = −0.425; *p* = 0.001) were associated with greater postoperative score increase. **Conclusions:** In this retrospective paired comparative study, erectile function was evaluated in male patients undergoing elective inguinal hernia repair using paired preoperative and sixth-month postoperative IIEF-5 scores. Erectile function appeared to be largely preserved after surgery, and the overall postoperative increase in IIEF-5 score was statistically significant but small in magnitude. No significant difference in IIEF-5 score change was observed between laparoscopic and open Lichtenstein repair. Scrotal hernia status and lower baseline IIEF-5 scores were associated with greater postoperative IIEF-5 score increase; however, this subgroup finding should be interpreted as exploratory and requires confirmation in larger prospective cohorts. These findings may help provide more realistic expectations during preoperative counseling, while avoiding causal interpretation.

## 1. Introduction

Inguinal hernia repair is among the most commonly performed procedures in general surgery. Current guidelines support tension-free mesh-based repair as the standard approach for primary inguinal hernias [1]. Open Lichtenstein repair and laparoscopic techniques, including transabdominal preperitoneal repair (TAPP) and totally extraperitoneal repair (TEP), are widely used surgical options. The choice of technique is usually guided by postoperative pain, recurrence risk, complication profile, recovery time, return to daily activities, and quality-of-life outcomes [2,3]. However, erectile function and sexual health-related outcomes have received less attention in routine surgical evaluation. Existing evidence on this issue remains heterogeneous and partly inconsistent [4,5,6].

Hernia-related pain, swelling, discomfort, and impaired quality of life may negatively affect sexual activity and erectile function before surgery [7]. After inguinal hernia repair, erectile function may be influenced by early postoperative pain, edema, chronic groin pain, nerve irritation or injury, spermatic cord manipulation, and mesh-related fibrosis [8]. The ilioinguinal, iliohypogastric, and genital branches of the genitofemoral nerve are particularly relevant in this context. Laparoscopic repair may offer faster functional recovery because of reduced tissue trauma. Open repair, on the other hand, may involve greater exposure or manipulation of inguinal nerves and spermatic cord structures [9]. Scrotal hernias represent a distinct subgroup. More extensive involvement of the inguinoscrotal region, testicular discomfort, traction on spermatic cord structures, and psychosocial concerns may contribute to lower preoperative erectile function. Successful repair may therefore result in a more pronounced postoperative improvement in these patients [8].

The International Index of Erectile Function-5 (IIEF-5) is a practical and widely used tool for evaluating erectile function. It is easy to administer, validated in different languages and populations, and allows classification of erectile dysfunction severity according to the total score [10,11]. The IIEF-5 does not assess all components of male sexual health, such as libido, ejaculation, orgasmic function, sexual pain, or partner-related factors. Nevertheless, it provides a standardized and comparable measure of erectile function in clinical studies [12].

Studies evaluating erectile function after inguinal hernia repair have reported conflicting results. Some studies have suggested short-term advantages of laparoscopic repair, while others have found no significant difference between laparoscopic and open techniques [13,14]. Several methodological limitations may explain these inconsistencies, including small sample sizes, retrospective designs, absence of standardized preoperative assessment, variable follow-up intervals, and inadequate adjustment for confounding factors such as age, hernia defect size, baseline erectile function, and scrotal hernia status [15].

The present study evaluated changes in IIEF-5 scores in male patients undergoing elective unilateral inguinal hernia repair using a paired preoperative and postoperative comparative design. Preoperative erectile function was assessed within one month before surgery, and postoperative erectile function was reassessed at the sixth postoperative month. The primary aim was to compare changes in erectile function between laparoscopic TAPP/TEP repair and open Lichtenstein repair. Secondary aims were to evaluate the effect of scrotal hernia on postoperative IIEF-5 changes, identify independent predictors of erectile function improvement, and describe transitions between erectile dysfunction severity categories after surgery.

## 2. Materials and Methods

This retrospective paired comparative study included male patients who underwent elective repair for primary unilateral inguinal hernia at the Department of General Surgery, Ege University. Operations were performed between January 2019 and December 2023.

Male patients aged 18 years or older who underwent either laparoscopic transabdominal preperitoneal repair/totally extraperitoneal repair or open Lichtenstein repair were considered eligible. Patients were included if both preoperative and sixth-month postoperative IIEF-5 assessments were available and written informed consent had been obtained for the retrospective use of anonymized questionnaire responses and relevant clinical data. Preoperative erectile function was assessed using the International Index of Erectile Function-5 questionnaire during routine preoperative evaluation within one month before surgery. Postoperative erectile function was reassessed using the same questionnaire during the routine face-to-face outpatient visit at the sixth postoperative month. The sixth postoperative month was selected as the standardized follow-up time point because early postoperative factors such as pain, edema, wound healing, temporary activity restriction, and delayed resumption of sexual activity may transiently influence erectile function assessments. This time point also corresponded to the routine institutional follow-up schedule and allowed a medium-term evaluation after the early recovery phase.

Patients with recurrent or bilateral inguinal hernia, emergency surgery, a history of concomitant pelvic surgery, severe urological or neurological disease that could affect erectile function, advanced cardiovascular disease that could influence erectile function or surgical outcomes, advanced respiratory disease, end-stage renal failure requiring dialysis, advanced liver failure, or active malignancy were excluded. Patients who declined participation, did not provide written informed consent, could not be reached for postoperative assessment, or had incomplete questionnaire data were also excluded.

Among 159 eligible patients, 11 could not be reached and 22 declined to participate. Therefore, 126 patients were included in the final analysis. Demographic and clinical data, including age, surgical technique, hernia side, defect diameter, and the presence of scrotal hernia, were obtained from institutional surgical records. Surgical technique was categorized as laparoscopic repair or open Lichtenstein repair. All operations were performed by the same experienced hernia surgery team under the supervision of the same senior surgeon. Both laparoscopic and open repairs were conducted according to standardized institutional surgical protocols. This approach was intended to minimize surgeon-related variability in operative technique and perioperative management. The laparoscopic group included both transabdominal preperitoneal repair (TAPP) and totally extraperitoneal repair (TEP). Because of the limited number of patients in the laparoscopic subgroups, TAPP and TEP were pooled and analyzed as a single laparoscopic repair group. Routine tacker fixation was used in all laparoscopic procedures. The choice of surgical technique was not randomized. In routine clinical practice, the decision to perform laparoscopic or open repair was made through shared decision-making between the patient and the surgical team, taking into account clinical suitability for laparoscopy and general anesthesia, hernia characteristics, previous abdominal surgery, comorbid conditions, patient preference, and institutional/surgeon-related feasibility. All procedures were performed by the same experienced hernia surgery team under the supervision of the same senior surgeon, according to standardized institutional surgical protocols.

The IIEF-5 questionnaire consists of five items, each scored from 1 to 5. The total score ranges from 5 to 25, with higher scores indicating better erectile function. Erectile dysfunction severity was categorized according to the total score as follows: 5–7, severe erectile dysfunction; 8–11, moderate erectile dysfunction; 12–16, mild-to-moderate erectile dysfunction; 17–21, mild erectile dysfunction; and 22–25, no erectile dysfunction. Internal consistency of the IIEF-5 questionnaire was assessed using Cronbach’s alpha coefficient separately for the preoperative and postoperative assessments. Cronbach’s alpha was 0.91 for the preoperative assessment and 0.77 for the postoperative assessment.

Statistical analyses were performed using IBM SPSS Statistics for Windows, Version 26.0 (IBM Corp., Armonk, NY, USA). Continuous variables were expressed as mean ± standard deviation or median with minimum–maximum values, depending on distribution. Categorical variables were presented as numbers and percentages. The Shapiro–Wilk test was used to assess normality.

Preoperative and sixth-month postoperative IIEF-5 scores were compared using paired-samples t-test for normally distributed variables and Wilcoxon signed-rank test for non-normally distributed variables. Changes in IIEF-5 scores were calculated by subtracting the preoperative score from the postoperative score. Between-group comparisons of IIEF-5 score changes were performed using the independent-samples t-test or Mann–Whitney U test, as appropriate. Categorical variables were compared using the chi-square test or Fisher’s exact test. Changes in the presence or absence of erectile dysfunction before and after surgery were evaluated using McNemar’s test, while erectile dysfunction severity categories were summarized descriptively. Independent predictors of postoperative change in IIEF-5 score were evaluated using multivariate linear regression analysis. A *p* value of <0.05 was considered statistically significant.

The study was approved by the Ege University Clinical Research Ethics Committee as a retrospective paired observational study using previously collected routine clinical and IIEF-5 questionnaire data (protocol code: 25-11T/71; approval date: 6 November 2025). The ethics committee approved the retrospective analysis of anonymized clinical data and paired IIEF-5 questionnaire responses. Although the operations and routine clinical follow-up assessments were performed between January 2019 and December 2023, data extraction, anonymization, final dataset preparation, and statistical analyses for the present study were performed only after ethics committee approval. Written informed consent was obtained from all included participants for the retrospective use of their anonymized questionnaire responses and relevant clinical data for research and publication purposes. The study was conducted in accordance with the principles of the Declaration of Helsinki.

## 3. Results

A total of 126 male patients were included in the final analysis. Laparoscopic repair was performed in 59 patients (46.8%), including TAPP in 40 patients and TEP in 19 patients, while 67 patients (53.2%) underwent open Lichtenstein repair. The mean age of the overall cohort was 53.5 ± 13.2 years, and the mean hernia defect diameter was 2.23 ± 1.16 cm. Scrotal hernia was present in 14 patients (11.1%).

Baseline characteristics were comparable between the laparoscopic and open repair groups. In the open repair group, 33 patients had right-sided and 34 had left-sided hernias; in the laparoscopic group, 32 patients had right-sided and 27 had left-sided hernias. Scrotal hernia was observed in 5 patients (7.5%) in the open group and in 9 patients (15.3%) in the laparoscopic group. No statistically significant differences were observed between the groups in terms of age, defect diameter, preoperative IIEF-5 score, hernia side, or scrotal hernia status (all *p* > 0.05). Baseline demographic and clinical characteristics according to surgical technique are presented in Table 1.

Baseline comparability between the laparoscopic and open repair groups allowed a more balanced assessment of postoperative IIEF-5 changes according to surgical technique. In particular, similar preoperative IIEF-5 scores between the groups provided an appropriate basis for comparing changes in erectile function.

In the overall cohort, the mean IIEF-5 score increased from 22.85 ± 3.12 preoperatively to 23.35 ± 1.98 at the sixth postoperative month. The mean change was +0.50 ± 2.49 points, and this increase was statistically significant (*p* = 0.026). The mean IIEF-5 score change was +0.61 ± 2.60 in the laparoscopic group and +0.40 ± 2.41 in the open Lichtenstein group. However, the difference in score change between the two surgical techniques was not statistically significant (*p* > 0.05). Preoperative and sixth-month postoperative IIEF-5 scores and corresponding changes are summarized in Table 2.

In both surgical groups, mean IIEF-5 scores showed a slight postoperative increase. The mean increase was numerically greater in the laparoscopic group than in the open Lichtenstein group; however, the difference between techniques was not statistically significant. Therefore, surgical approach was not associated with a significant difference in postoperative erectile function change.

Age was additionally compared between patients with and without scrotal hernia. Patients with scrotal hernia were not younger than those without scrotal hernia, and no statistically significant age difference was observed between the groups (56.79 ± 11.79 vs. 53.15 ± 13.37 years, *p* = 0.332). Patients with scrotal hernia showed a markedly greater improvement in IIEF-5 scores. In this subgroup, the mean IIEF-5 score increased from 15.86 ± 4.74 preoperatively to 21.43 ± 2.44 at the sixth postoperative month, corresponding to a mean change of +5.57 ± 3.52 points. In contrast, patients without scrotal hernia showed no meaningful postoperative improvement, with a mean change of −0.13 ± 1.37 points. The difference in IIEF-5 score change between patients with and without scrotal hernia was statistically significant (*p* < 0.001). These findings are summarized in Table 3.

The lower baseline IIEF-5 scores observed in patients with scrotal hernia may partly explain the more pronounced postoperative improvement in this subgroup. This finding should be interpreted cautiously because the scrotal hernia subgroup was small and had substantially lower baseline IIEF-5 scores. Therefore, the observed improvement may partly reflect greater room for numerical recovery, regression to the mean, or baseline imbalance rather than a reliable functional effect of scrotal hernia repair.

When erectile dysfunction status was dichotomized as absent or present, the number of patients without erectile dysfunction increased from 114 preoperatively to 117 at the sixth postoperative month. However, this change was not statistically significant (McNemar test, *p* = 0.453). The distribution of erectile dysfunction severity categories was also evaluated descriptively. Preoperatively, 114 patients had no erectile dysfunction, 3 had mild erectile dysfunction, 7 had mild-to-moderate erectile dysfunction, and 2 had moderate erectile dysfunction. At the sixth postoperative month, 117 patients had no erectile dysfunction, 6 had mild erectile dysfunction, 3 had mild-to-moderate erectile dysfunction, and no patient had moderate erectile dysfunction. These data are summarized in Table 4.

The postoperative increase in total IIEF-5 score reached statistical significance in the overall cohort; however, this numerical improvement was not accompanied by a significant change in erectile dysfunction status when evaluated as a binary outcome. This finding likely reflects the limited magnitude of individual score changes, which were generally insufficient to shift patients across established erectile dysfunction severity thresholds.

In multivariate linear regression analysis, scrotal hernia was associated with a greater postoperative increase in IIEF-5 score (β = +2.41; *p* = 0.041), whereas higher baseline IIEF-5 scores were associated with less postoperative improvement (β = −0.425; *p* = 0.001). Surgical technique, age, defect diameter, and hernia side were not independently associated with postoperative IIEF-5 change. Overall, these findings indicate that postoperative erectile function improvement was more closely related to baseline erectile function and scrotal hernia status than to the choice of surgical technique.

## 4. Discussion

In the present study, erectile function was assessed using paired preoperative and sixth-month postoperative IIEF-5 scores in male patients undergoing elective inguinal hernia repair. Although total IIEF-5 scores increased significantly in the overall cohort, this improvement was modest and did not differ significantly between laparoscopic and open Lichtenstein repair. The largest numerical improvement was observed in patients with scrotal hernia; however, this subgroup included only 14 patients and had lower baseline IIEF-5 scores. Therefore, this finding should be interpreted as hypothesis-generating rather than as evidence that scrotal hernia repair reliably improves erectile function. In multivariate analysis, scrotal hernia status and lower baseline IIEF-5 score were associated with greater postoperative improvement, whereas surgical technique was not. These findings suggest that postoperative erectile function outcomes are influenced more by baseline functional status and hernia-related characteristics than by the operative approach alone.

Previous studies comparing laparoscopic TAPP/TEP and open Lichtenstein repair have produced inconsistent results regarding postoperative erectile function. While some studies have reported short-term advantages after laparoscopic repair, others have found no significant difference between techniques [8,16]. The absence of a significant between-group difference in our cohort is consistent with this heterogeneity. Potential mechanisms include nerve exposure or manipulation during open repair, as well as preperitoneal dissection and tacker fixation during laparoscopic repair, all of which may contribute to postoperative pain or discomfort in selected patients [17,18]. In addition, mesh properties, fixation technique, and nerve-sparing principles have been emphasized as important factors in the development of chronic groin pain, which may indirectly affect erectile function and other sexual health-related outcomes [19,20]. Nevertheless, these factors did not appear to produce a measurable difference in sixth-month IIEF-5 score changes between the two surgical approaches in the present study. Therefore, the comparison between laparoscopic and open repair should be interpreted as an observational comparison rather than evidence of equivalence between techniques.

The greater numerical improvement observed in patients with scrotal hernia should be interpreted cautiously. Although scrotal hernias may plausibly affect preoperative erectile function through traction or compression of spermatic cord structures, testicular discomfort, local temperature changes, cosmetic concerns, and psychosocial distress [21,22,23], the present subgroup included only 14 patients. Therefore, the observed effect size may be unstable and vulnerable to regression to the mean, baseline imbalance, and ceiling effects. In particular, patients with scrotal hernia had substantially lower baseline IIEF-5 scores, whereas patients without scrotal hernia had high baseline scores and limited room for further numerical improvement. Thus, the present findings should not be interpreted as evidence that scrotal hernia repair reliably improves erectile function. Rather, patients with scrotal hernia and lower baseline IIEF-5 scores may represent a subgroup with greater potential for postoperative improvement. This hypothesis requires confirmation in larger prospective cohorts with more balanced baseline erectile function and clinically meaningful change thresholds [24].

Male sexual health is a multidimensional concept that includes not only erectile function but also libido, ejaculation, orgasmic function, pain during sexual activity, and partner-related factors. In this context, the IIEF-5 provides a practical and validated measure of erectile function, but it does not fully capture all domains of postoperative sexual health. Therefore, the findings of the present study should be interpreted primarily as changes in erectile function rather than global sexual function. Several biological and psychosocial mechanisms may influence sexual health after inguinal hernia repair. Chronic groin pain, allodynia or hyperalgesia, nerve irritation or neuropathy, postoperative edema, and mesh-related fibrosis may interact with anxiety, body image concerns, and sexual self-confidence [25]. Although laparoscopic repair is generally associated with less anterior groin dissection and faster functional recovery, preperitoneal mesh placement and fixation may contribute to chronic pain or discomfort in selected patients [26,27]. In open Lichtenstein repair, nerve identification and preservation, as well as selective neurectomy when clinically indicated, may reduce the risk of chronic pain; however, their direct impact on postoperative erectile function remains unclear [28,29].

Beyond local surgical, mechanical, pain-related, and psychosocial factors, erectile function is also influenced by vascular, endothelial, inflammatory, and systemic mechanisms. Recent literature on vasculogenic erectile dysfunction has emphasized the interaction between immune modulation, vascular repair, endothelial function, and erectile physiology, including the potential role of immune-cell-based regenerative approaches. Although the present study did not evaluate endothelial function, vasculogenic mechanisms, inflammatory biomarkers, or regenerative therapies, this broader biological framework may be relevant when interpreting postoperative IIEF-5 changes, particularly in patients with impaired baseline erectile function. Therefore, future studies evaluating sexual function after inguinal hernia repair may benefit from incorporating vascular, metabolic, inflammatory, and systemic variables in addition to surgical and pain-related factors [30].

The main strengths of this study are the paired preoperative and sixth-month postoperative assessment of IIEF-5 scores in the same patients, the satisfactory internal consistency of the IIEF-5 questionnaire in both assessment periods, and the use of multivariate analysis to adjust for clinically relevant variables, including scrotal hernia status, age, defect diameter, surgical technique, and baseline IIEF-5 score. In addition, the comparable baseline characteristics of the laparoscopic and open repair groups, particularly age, defect diameter, and preoperative IIEF-5 scores, supported a more balanced comparison of postoperative erectile function changes between surgical techniques.

This study has several limitations. First, although both preoperative and sixth-month postoperative IIEF-5 assessments were performed during face-to-face clinical visits, erectile function was evaluated using a patient-reported questionnaire rather than a multidimensional sexual health assessment. Therefore, the findings should be interpreted specifically as changes in erectile function measured by IIEF-5, rather than as a comprehensive evaluation of overall male sexual health. Second, postoperative erectile function was assessed at a single standardized time point. As a result, early postoperative changes, recovery patterns over time, and longer-term functional outcomes beyond six months could not be evaluated. Third, variables that may influence postoperative erectile function, including postoperative pain intensity, chronic groin pain, analgesic use, sexual pain, and time to resumption of sexual activity, were not systematically recorded. Another important limitation is the small size of the scrotal hernia subgroup. Because only 14 patients had scrotal hernia, the estimated improvement in IIEF-5 score may be unstable and susceptible to regression to the mean, baseline imbalance, and ceiling effects. Therefore, this subgroup finding should be considered exploratory and hypothesis-generating. The absence of these data limits the ability to clarify the mechanisms underlying postoperative IIEF-5 changes. In addition, potential confounders such as hormonal status, psychiatric factors, medication use, smoking status, diabetes severity, and other comorbid conditions could not be fully incorporated into the multivariate model. Nevertheless, patients with severe urological, neurological, cardiovascular, respiratory, renal, hepatic, or malignant diseases were excluded, which may have improved the internal comparability of the study population. Although all procedures were performed within the same experienced surgical team under the supervision of the same senior surgeon, individual surgeon-related variability cannot be completely excluded in a retrospective study. In addition, operative time, detailed intraoperative technical variations, and minor postoperative complications were not analyzed as independent variables. Therefore, the potential influence of these factors on postoperative erectile function could not be fully assessed. Another limitation is the non-randomized selection of surgical technique. Although laparoscopic and open repairs were performed by the same experienced hernia surgery team under the supervision of the same senior surgeon, the choice of technique was based on routine clinical decision-making rather than random allocation. Therefore, selection bias cannot be fully excluded. In addition, operative time, cost-related variables, patient satisfaction, and detailed surgeon-specific or intraoperative technical factors were not systematically analyzed. The absence of these variables limits the ability to fully assess how procedural factors may have influenced postoperative erectile function outcomes. Propensity score matching was not performed because of the limited sample size and the risk of substantially reducing the effective cohort, particularly in subgroup analyses such as scrotal hernia. Instead, baseline comparability between laparoscopic and open repair groups was assessed for clinically relevant variables, including age, hernia side, defect diameter, scrotal hernia status, and preoperative IIEF-5 score, and multivariate linear regression was used to adjust for relevant covariates. Nevertheless, residual selection bias cannot be fully excluded due to the retrospective and non-randomized design. Finally, laparoscopic TAPP and TEP procedures were pooled and analyzed as a single laparoscopic repair group. However, these techniques differ in surgical access, peritoneal handling, dissection plane, and potentially postoperative pain profile. Therefore, pooling TAPP and TEP may have obscured technique-specific effects on postoperative erectile function, particularly with respect to pain- or nerve-related mechanisms. Because of the limited number of patients in the TAPP and TEP subgroups, separate statistical comparisons were not performed. Future studies with larger cohorts should evaluate TAPP and TEP separately.

From a clinical perspective, these findings may support more individualized preoperative counseling. Erectile function was largely preserved after both laparoscopic and open inguinal hernia repair, with no significant difference between techniques at the sixth postoperative month. Therefore, expectations regarding postoperative erectile function alone should not determine the choice of surgical approach. Patients with scrotal hernia and those with lower baseline IIEF-5 scores appear to have greater potential for postoperative improvement, and this may be discussed during counseling. Nevertheless, individual risk factors, including comorbidities, medication use, and pain-related symptoms, should be considered. Future studies should use prospective designs with multidimensional assessments, including IIEF-15, pain scores, quality-of-life measures, and partner-reported outcomes. Separate evaluation of TAPP and TEP, mesh type, fixation method, and clinically meaningful change thresholds may further clarify the determinants of postoperative erectile function, particularly in patients with scrotal hernia.

## 5. Conclusions

In this retrospective paired comparative study, erectile function was evaluated in male patients undergoing elective inguinal hernia repair using paired preoperative and sixth-month postoperative IIEF-5 scores. Erectile function appeared to be largely preserved after surgery, and the overall postoperative increase in IIEF-5 score was statistically significant but small in magnitude. No significant difference in IIEF-5 score change was observed between laparoscopic and open Lichtenstein repair. Scrotal hernia status and lower baseline IIEF-5 scores were associated with greater postoperative IIEF-5 score increase; however, this subgroup finding should be interpreted as exploratory and requires confirmation in larger prospective cohorts. These findings may help provide more realistic expectations during preoperative counseling and support individualized surgical decision-making without implying a causal effect.

## Figures and Tables

**Table 1:** Baseline characteristics of patients according to surgical technique.

	Open (Lichtenstein)	Laparoscopic	*p* Value
Age (mean ± SD)	54.84 ± 12.87	52.10 ± 13.55	0.248
Defect diameter, cm (mean ± SD)	2.16 ± 1.08	2.33 ± 1.26	0.415
Preoperative IIEF-5 (mean ± SD)	23.25 ± 2.66	22.39 ± 3.55	0.122
Hernia Side (n) Right Left	33 34	32 27	0.574
Scrotal Hernia (n)	5 (7.5%)	9 (15.3%)	0.165

**Table 2:** Preoperative and postoperative IIEF-5 scores and changes according to surgical technique and in the overall cohort.

Group	n	Preoperative Mean ± SD	Postoperative Mean ± SD	Mean Change ± SD	Within-Group *p* Value
Overall cohort	126	22.85 ± 3.12	23.35 ± 1.98	0.50 ± 2.49	0.026
Laparoscopic	59	22.39 ± 3.55	23.00 ± 2.22	0.61 ± 2.60	0.077
Open (Lichtenstein)	67	23.25 ± 2.66	23.66 ± 1.69	0.40 ± 2.41	0.175

Between-group comparison of mean change: *p* = 0.643. Within-group comparisons were performed using paired-samples *t*-test. Between-group comparisons of change scores were performed using independent-samples *t*-test.

**Table 3:** Preoperative and postoperative changes in IIEF-5 scores according to the presence of scrotal hernia.

Group	n	Preoperative Mean ± SD	Postoperative Mean ± SD	Mean Change ± SD	Within-Group *p* Value
Scrotal hernia present	14	15.86 ± 4.74	21.43 ± 2.44	5.57 ± 3.52	<0.001
No scrotal hernia	112	23.72 ± 1.19	23.59 ± 1.78	−0.13 ± 1.37	0.304

Between-group comparison of mean change: *p* < 0.001. Within-group comparisons were performed using paired-samples *t*-test. Between-group comparisons of change scores were performed using independent-samples *t*-test.

**Table 4:** Distribution of erectile dysfunction severity before and after surgery.

Category	Preoperative (n)	Postoperative (n)
No ED	114	117
Mild ED	3	6
Mild-to-Moderate ED	7	3
Moderate ED	2	0

## Data Availability

The data supporting the findings of this study are not publicly available due to privacy and ethical restrictions but may be made available from the corresponding author upon reasonable request and with permission from the relevant institutional authorities.
